# Factors associated with metabolic syndrome among adult residents in Dalian: a nested case-control study

**DOI:** 10.3389/fendo.2025.1559176

**Published:** 2025-07-17

**Authors:** Rong Rong, Lan Luo, Xinyu Li, Zhengnan Gao

**Affiliations:** Department of Endocrinology and Metabolism, Central Hospital of Dalian University of Technology, Dalian Municipal Central Hospital, Dalian, Liaoning, China

**Keywords:** metabolic syndrome, risk factors, nested case-control study, fresh juice, beef and mutton, sleep duration, adult residents in Dalian, body mass index

## Abstract

**Objective:**

This study aimed to investigate risk factors for metabolic syndrome (MS) among adult residents in Dalian, Liaoning Province, China, using a nested case-control design.

**Methods:**

Adult participants from Dalian who took part in both baseline and follow-up phases of the Risk Evaluation of Cancers in Chinese Diabetic Individuals: A Longitudinal (REACTION) Study were evaluated through standardized questionnaires, physical examinations, and biochemical analyses. A total of 536 individuals diagnosed with MS were matched in a 1:4 ratio to 2,144 controls based on comparable demographic and clinical characteristics. Group differences were assessed via t-tests, rank sum tests, and χ² tests. Multivariate conditional logistic regression was applied to identify risk factors for MS.

**Results:**

(1) The case group demonstrated significantly higher values for body weight(67.42 ± 9.77 vs. 62.39 ± 9.31, *P*<0.001), body mass index (BMI) (25.99 ± 3.36 vs 24.00 ± 3.14, *P*<0.001), hip circumference (HC) (100.72 ± 6.47 vs 97.84 ± 6.38, *P*<0.001), homeostatic model assessment for insulin resistance (HOMA−IR) (2.27 ± 1.19 vs 1.70 ± 0.92, *P*<0.001),total cholesterol (TC) (5.54 ± 1.08 vs 5.40 ± 0.97, *P*=0.003), low-density lipoprotein cholesterol (LDL-C) (3.38(2.79,3.96) vs 3.17(2.67,3.71), *P*<0.001), alanine aminotransferase (ALT) (16.00(13.00,21.00) vs 15.00(11.00,19.00), *P*<0.001), gamma-glutamyl transferase (GGT) (22.00(17.00,33.00) vs 18.00(14.00,27.00), *P*<0.001), serum uric acid (UA) (303.50(263.00,355.00) vs 281.00(245.00,325.00), *P*<0.001), glycosylated hemoglobin (HbA1c) (5.93 ± 0.88 vs 5.75 ± 0.68, *P*<0.001), and fasting insulin (FINS) (8.05(5.90,10.70) vs 6.15(4.60,8.30), *P*<0.001) (2). Higher prevalence rates were also observed for coronary heart disease (4.86% vs 2.87%, *P*=0.020), habitual snoring (66.53% vs 54.96%, *P*<0.001), and consumption of fresh juice (17.99% vs 13.12%, *P*=0.004), beef and mutton (78.42% vs 74.07%, *P*=0.038), and soda the case group (20.15% vs 16.32%, *P*=0.049). Meanwhile, lower participation in aerobic activities(1.20% vs 2.92%, *P*=0.030) and shorter average daily sleep duration (7.55 ± 1.02 vs 7.69 ± 1.17, *P*=0.028) were noted in the case group (3). Regression analysis identified longer average daily sleep duration as a protective factor(OR=0.844, 95%CI: 0.761-0.936, *P*=0.001), while fresh juice intake(OR=1.846, 95%CI: 1.315-2.592, *P*<0.001), beef and mutton consumption(OR=1.282, 95%CI:1.007-1.632, *P*=0.044), LDL-C(OR=1.409, 95%CI: 1.245-1.595, *P*<0.001), GGT(OR=1.004, 95%CI: 1.001-1.008, *P*=0.017), UA(OR=1.005, 95%CI: 1.003-1.007, *P* < 0.001), HOMA-IR (OR=1.464, 95%CI: 1.313-1.633, *P* < 0.001), HC(OR=1.030, 95%CI: 1.007-1.053, *P* = 0.009), and BMI(OR=1.118, 95%CI: 1.064-1.174, *P* < 0.001)were significant risk factors.

**Conclusion:**

LDL-C, GGT, UA, HOMA-IR, HC, BMI, daily sleep duration, and consumption of beef and mutton, and fresh juice were strongly associated with the incidence of MS among adult residents in Dalian.

## 1 Introduction

Metabolic syndrome (MS) is a clinical entity characterized by a cluster of abdominal obesity, hyperglycemia (diabetes or impaired glucose tolerance), dyslipidemia (elevated triglycerides and/or reduced high-density lipoprotein levels), and hypertension—factors that collectively exert a substantial influence on systemic health. It comprises a constellation of metabolically interrelated risk elements (1), and is a multifaceted pathophysiological condition primarily stemming from an imbalance in caloric intake and energy expenditure, yet it is also modulated by factors such as an individual’s genetic/epigenetic constitution and lifestyle behaviors. The pathogenesis of MS is mainly mediated by increased free fatty acids leading to insulin resistance and chronic low-grade inflammation induced by pro-inflammatory cytokines (2). Over recent decades, the global incidence of MS has markedly increased, now affecting nearly one-quarter of the global population, which translates to over 1 billion individuals (3). Its treatability remains uncertain, combination of drug therapy and dietary adjustments, could be helpful in the prevention and management of MS (2). In China, rapid economic expansion accompanied by shifts in dietary patterns and lifestyle behaviors has further intensified the MS burden. Current research estimates that 19.58% of the Chinese population is affected by MS (4), with prevalence rates surging to 36.9% among the elderly demographic (5). MS has attracted much attention from scholars since it was proposed. Its high incidence of endpoint events, especially cardiovascular and cerebrovascular events, has become the first of the three causes of death, which seriously threatens human health. Research on the risk factors of metabolic syndrome can not only further explore its formation mechanism, but also accelerate the drug development process of related targets, timely urge people to improve their lifestyles, and enhance the health awareness of the whole population, which is of great significance for the prevention and treatment of MS. While factors such as age, body mass index (BMI), and insulin resistance are consistently recognized as key contributors, other risk factors remain unclear or yield inconsistent associations across different populations and geographical regions. A study conducted among elderly individuals in Shenzhen, China, identified regular rice consumption as a potential protective factor against MS, while reporting no significant association between alcohol intake and MS risk (6). In contrast, research involving Swedish adults suggested a possible protective effect of alcohol consumption for individuals with MS (7). Meanwhile, findings from a Korean cohort indicated that high rice intake may elevate the risk of abdominal obesity, a condition closely linked to the pathogenesis and progression of MS (8). As a historically significant coastal city, Dalian exhibits distinct dietary customs and lifestyle patterns. The city’s rapid socioeconomic development has led to an increasingly fast-paced lifestyle, contributing to a rise in metabolic disorder-related conditions. A cross-sectional study in adult residents of Shenzhen, a coastal city in China, has shown that significant differences were found in MS groups with different sociodemographic or other characteristics, such as age, serum uric acid(UA) levels, gender, smoking status, drinking status, marital status, BMI, and educational level, and increased UA levels were positively associated with the prevalence of MS and its components (9). Despite these trends, investigations into MS risk factors within the Dalian population remain lacking. Accordingly, this study adopted a nested case-control design to identify risk factors for MS among adult residents of Dalian. We hypothesize that specific dietary habits (e.g., consumption of fresh juice, beef and mutton, and soda), lifestyle factors (e.g., sleep duration, aerobic activities, and smoking), basic information (e.g., diseases history and anthropometric assessments), and relevant biochemical markers (e.g., LDL-C, GGT, UA, HOMA-IR) are associated with an increased risk of MS among adult residents in Dalian.

The nested case-control design, an advanced epidemiological methodology, integrates the methodological rigor of cohort studies with the efficiency of case-control frameworks. It is based on the follow-up observation of a pre-determined cohort, and then the design concept of case-control studies (mainly matching case-control studies) is applied for research and analysis, integrates the strengths of cohort and case-control designs. This approach improves research efficiency and cost management, while offering greater statistical robustness and diagnostic precision relative to traditional case-control models (10). Currently, this method is widely used in medical scientific research.

Utilizing data from the Risk Evaluation of Cancers in Chinese Diabetic Individuals: A Longitudinal (REACTION) Study, a follow-up cohort was established to investigate MS among adult residents in the Dalian community. Through a matched nested case-control framework, the study assessed the associations between the onset of MS and a comprehensive range of biochemical indicators, demographic characteristics, medical history, and lifestyle variables—including dietary patterns, physical activity, and habitual behaviors. The objective was to optimize early detection of risk factors, support timely intervention strategies, and reduce MS incidence, thereby minimizing its broader personal, familial, and social burden.

## 2 Materials and methods

### 2.1 Study participants

The REACTION Study, a multicenter prospective cohort investigation, enrolled Chinese adults aged ≥40 years from the Dalian community who participated in the baseline epidemiological survey at the Dalian subcenter between August and December 2011(n=10208, 2807 males and 7401 females), followed by re-evaluation from July to December 2014(n=5354, 1369 males and 3985 females). Longitudinal data were obtained through standardized physical examinations, biochemical assessments, and structured data collection at both time points. A nested case-control design was employed in this study. Each incident MS case identified within the cohort was matched to one or more controls who remained free of MS at the time of diagnosis. Case group: A total of 536 cases newly diagnosed MS during the follow-up period (2014) from the study population were included, as per the 2020 Chinese Diabetes Society diagnostic criteria (see Section 2.2). Control group: Controls were selected from the same cohort among individuals who remained free of MS at follow-up (2014). To minimize confounding, controls were matched to cases in a 4:1 ratio based on the following criteria:1) Gender: Exact matching (male/female). 2) Age: ± 3 years from the cases’ age at baseline. Controls were required to have completed both baseline and follow-up assessments, with no missing data on MS diagnostic components. Matching was performed using a stratified random sampling approach within each gender-age stratum to avoid overmatching. Exclusion criteria included missing data on biochemical or physical examinations(n=7), a prior diagnosis of MS(n=2367), clinically relevant cardiac, hepatic, or renal dysfunction(n=6), or chronic glucocorticoid therapy(n=2). The protocol was approved by the REACTION Study Ethics Committee [Approval No (2011). LLS No (14).], and all participants provided written informed consent.

### 2.2 Study methods

(1) Prior to survey implementation, the research personnel—including endocrinologists, postgraduate trainees, and nurses from Dalian Municipal Central Hospital Affiliated to Dalian University of Technology—received standardized training conducted by Ruijin Hospital, Shanghai Jiaotong University School of Medicine. All questionnaire data collection and anthropometric measurements were performed by trained staff according to a standard protocol. Informed consent was obtained from all enrolled community residents before data collection commenced.

(2) Baseline characteristics and outcome indicators were systematically collected. Participants completed structured questionnaires, underwent physical assessments, and provided venous blood specimens. Documented variables included demographic data (gender, age), individual and familial disease histories, marital and educational status, pharmacological treatments, sleep patterns, emotional well-being, and lifestyle parameters including dietary intake, physical activity, and daily routines. Clinical measurements included systolic and diastolic blood pressure (SBP and DBP), heart rate (HR), height, weight, waist and hip circumference (HC), and BMI was subsequently derived. Blood sampling was performed in the morning after an overnight fast of at least 8-14h. Fasting plasma glucose(FPG), 2 hours plasma glucose(2hPG), glycosylated hemoglobin (HbA1c), fasting insulin (FINS), and several biochemical markers—alanine aminotransferase (ALT), Aspartate aminotransferase(AST), gamma-glutamyl transferase (GGT), serum creatinine(Scr), total cholesterol (TC), Triglyceride (TG), low-density lipoprotein cholesterol (LDL-C), High density lipoprotein cholesterol (HDL-C), UA, triiodothyronine (FT3), free thyroxin (FT4), thyroid-stimulating hormone (TSH), thyroglobulin antibodies (TgAb), and thyroid peroxidase antibodies (TPOAb)—were measured. In addition, all participants underwent an oral glucose tolerance test(OGTT).

(3) Biochemical Evaluation: Fasting venous blood was collected in standard biochemical tubes, centrifuged immediately(within 2 hours), aliquoted into 0.5‐mL Eppendorf tubes, stored at -20°C, and transported within 3 weeks under cold-chain conditions to Ruijin Hospital, Shanghai Jiaotong University School of Medicine, Shanghai Institute of Endocrine and Metabolic Diseases, which is certified by the College of American Pathologists, for centralized analysis. Levels of Scr, TC, LDL-C, HDL-C, and TG were measured on an autoanalyzer (c16000 system, ARCHITECT ci16200 analyzer; Abbott Laboratories, Lake Bluff, IL) in the central laboratory. FINS was measured with chemiluminescent immunoassay (i2000SR system, Architect ci16200 analyzer; Abbott Laboratories). The levels of HbA1c were assayed by means of high‐performance liquid chromatography method (Variant II and D‐10 Systems; Bio‐Rad, Hercules, CA).FPG and 2hPG levels were measured from NaF-anticoagulated blood using the hexokinase method on an automated biochemical analyzer (ADVIA 2400 system). Homeostatic model assessment of insulin resistance (HOMA-IR)was calculated using the mathematical formula as follows: HOMA-IR = FPG (mmol/L) × FINS (µU/mL)/22.5 (11).UA concentrations were determined from fasting venous samples using the uricase colorimetric method on the ADVIA Chemistry XPT system. Thyroid function was evaluated via chemiluminescence immunoassay (Abbott I2000, Abbott reagent).

Data Collection: Epidemiological data were collected via one-on-one questionnaires, encompassing sociodemographic characteristics, lifestyle factors, and medical histories. The REACTION study questionnaire was developed through a systematic review of questionnaires related to MS, diabetes, and cancer both domestically and internationally(e.g., the International Physical Activity Questionnaire, IPAQ, Food Frequency Questionnaire, FFQ), and a working group composed of experts from multiple disciplines including endocrinology, epidemiology, and nutrition decided the content and structure of the questionnaire. Information on intensity, duration, and frequency of physical activity was gathered using the short form of the IPAQ. In the dietary section of the questionnaire, data were obtained regarding usual dietary intake over the past 12 months. The questionnaire was designed to capture information on frequency and quantity of major food items such as red meat, fruits and vegetables, dairy, and Chinese traditional food such as pickles and salty vegetables. The questionnaire has previously been evaluated and validated in other cohort studies (12–14).

Anthropometric assessments followed standardized procedures: weight was measured in the morning following an overnight fast, and height was recorded with participants standing upright, feet together, and arms relaxed. Height and weight were measured with participants wearing light‐weight clothes and no shoes. BMI was calculated by dividing weight (in kilograms) by weight (in meters) squared. Blood pressure and HR were measured at 5-minute intervals on the non-dominant arm in a resting state, with the mean of three readings recorded (1 mmHg = 0.133 kPa), using an automated electronic device (Omron Model HEM‐725 FUZZY; Omron Co, Dalian, China). Waist circumference(WC)was assessed at the midpoint between the lower rib and the anterior superior iliac spine, with participants standing upright, feet 25–30 cm apart, and breathing normally. HC was measured at the maximal circumference of the hips while standing, with legs together and arms relaxed.

**Figure 1:**
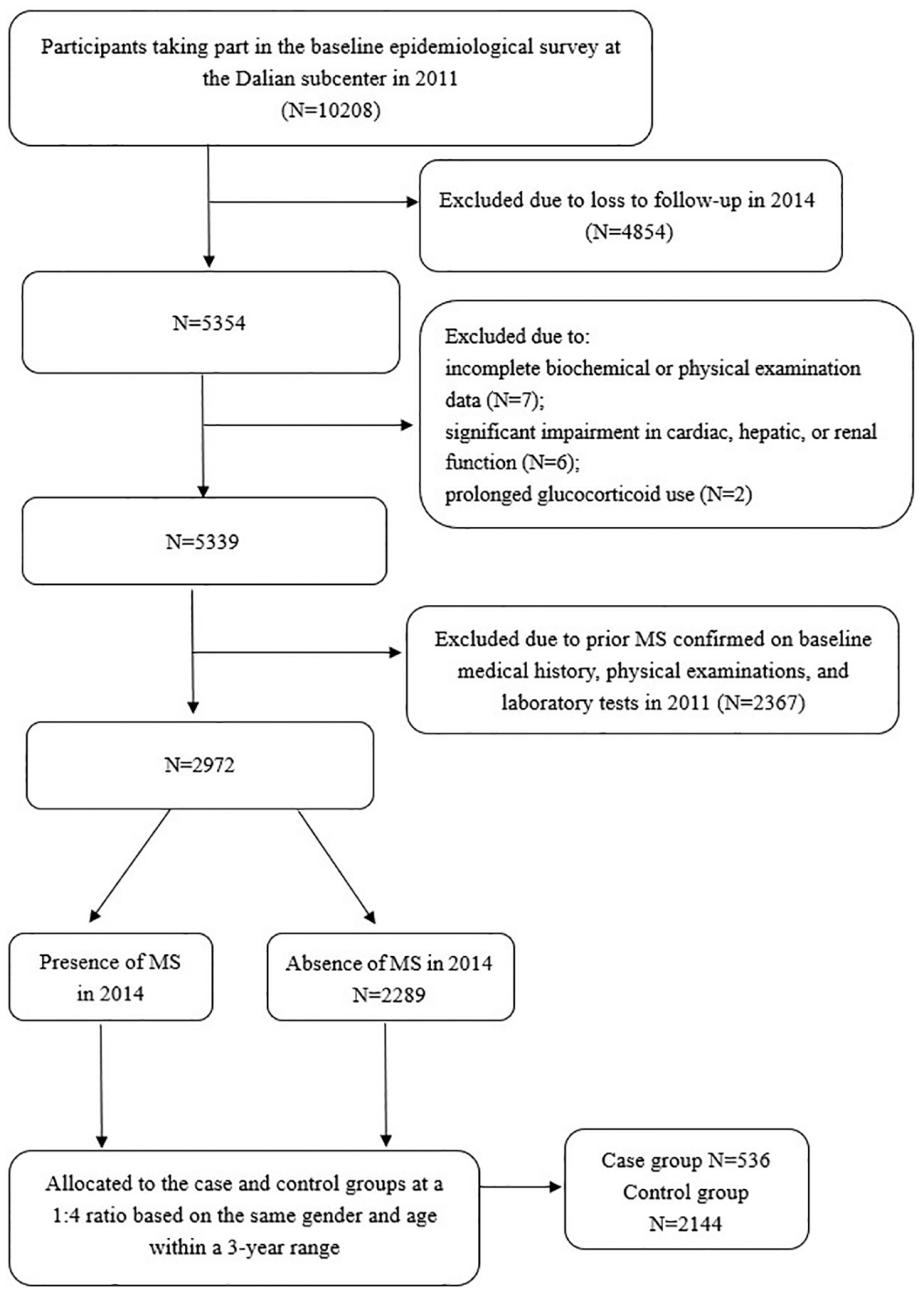
Flowchart of the study enrollment.

(4) Diagnostic and allocation criteria (1): MS diagnostic criteria: In accordance with the 2020 Guidelines of the Chinese Diabetes Society for the Prevention and Treatment of Type 2 Diabetes, a diagnosis of MS was established when at least three of the following five conditions were met: 1) Abdominal obesity, defined by a waist circumference ≥ 90 cm in men or ≥ 85 cm in women; 2) Hyperglycemia, determined by FPG ≥ 6.1 mmol/L and/or 2hPG ≥ 7.8 mmol/L, or a documented history of diabetes under treatment; 3) Hypertension, defined by blood pressure ≥ 130/85 mmHg, or a history of hypertension with ongoing treatment; 4) Elevated fasting triglycerides (≥ 1.70 mmol/L); 5) Decreased fasting HDL-C (< 1.04 mmol/L) (2). Case and control groups: A nested case-control design was employed. Each incident MS case identified within the cohort was matched to one or more controls who remained free of MS at the time of diagnosis. Ultimately, 2,680 participants (605 males and 2075 females) were included in the final analysis (**Figure 1**). A total of 536 newly diagnosed MS cases from the study population were included. A matching ratio of 1:4 was applied, with 2144 subjects without MS selected as controls. The controls were matched by gender and age, ensuring an age difference of less than 3 years.

### 2.3 Statistical methods

Statistical analyses were performed using SPSS 27.0. The distribution of measurement data was first evaluated; data conforming to normal distribution were presented as mean ± standard deviation (SD), whereas those deviating from normality were expressed as M *(Q1, Q3)*. Group comparisons for continuous variables employed the *t*-test when normality was verified by *P–P* plots, and the rank sum test for non-normally distributed data. Significance was defined as *P* < 0.05. Categorical variables were summarized as counts (%), with comparisons between groups conducted using the χ² test under the same significance criterion. Variables identified as significant in univariate analysis were incorporated into a multivariate conditional logistic regression model. A 1:4 matched conditional logistic regression (forward LR method) was used to identify risk factors for MS, with entry and removal criteria set at α = 0.05 and α = 0.10, respectively. A two-tailed *P* < 0.05 was considered indicative of statistical significance. OR value: >1 indicates risk factor, = 1 indicates no association, <1 indicates protective factor. 95% CI: includes1 indicates no statistical significance, excludes 1 indicates statistically significant. A *t*-test is a statistical hypothesis test used to determine whether there is a significant difference between the means of two groups or between a sample mean and a known population mean, and the data should be (approximately) normally distributed. The rank sum test, is a non-parametric statistical method used to compare two independent or paired samples when the data do not follow a normal distribution. The χ² test is a statistical hypothesis test used to examine the association between categorical variables or to assess how well observed data fit an expected distribution, and the data must be in frequency counts. Conditional logistic regression is a specialized regression analysis method designed for matched or stratified data, commonly employed in matched case-control studies. Its fundamental principle involves using conditional likelihood functions to eliminate the effects of confounding factors, thereby enabling more accurate estimation of the association between exposure variables and outcomes. The forward LR (likelihood ratio) method represents a variable selection strategy that progressively incorporates statistically significant variables into the model based on likelihood ratio tests, optimizing the model’s goodness-of-fit.

## 3 Results

### 3.1 Comparison of demographic and clinical data between groups

#### 3.1.1 Baseline demographic characteristics

The case group (n=536, males=121,females=415) and control group (n=2144,males=484,females=1660) were well-matched in terms of age (56.34 ± 7.41 vs 56.26 ± 7.46 years, *P*=0.819) and sex distribution sex distribution (22.57% male in both groups) (**Table 1**).

**Table 1:** Comparison of demographic and clinical data between groups.

Outcome measures	Case group (n=536)	Control group (n=2144)	*t/*Z value	*P* value
Age	56.34 ± 7.41	56.26 ± 7.46	0.229	0.819
Male [n (%)]	121 (22.57%)	484 (22.57%)		
Female [n (%)]	415 (77.43%)	1660 (77.43%)		
Height (cm)	160.95 ± 7.25	160.99 ± 7.66	-0.113	0.910
Weight (kg)	67.42 ± 9.77	62.39 ± 9.31	11.074	<0.001
BMI (kg/m^2^)	25.99 ± 3.36	24.00 ± 3.14	12.935	<0.001
HC (cm)	100.72 ± 6.47	97.84 ± 6.38	9.383	<0.001
HR (bpm)	78.61 ± 11.08	78.13 ± 11.40	1.023	0.307
HOMA-IR	2.27 ± 1.19	1.70 ± 0.92	12.185	<0.001
TC (mmol/L)	5.54 ± 1.08	5.40 ± 0.97	2.963	0.003
LDL-C (mmol/L)	3.38 (2.79,3.96)	3.17 (2.67,3.71)	-5.133	<0.001
Scr (umol/L)	63.30 (57.73,70.40)	62.55 (57.70,68.88)	-1.699	0.089
ALT (U/L)	16.00 (13.00,21.00)	15.00 (11.00,19.00)	-5.299	<0.001
AST (U/L)	21.00 (18.00,25.00)	21.00 (18.00,24.00)	-0.954	0.340
GGT (U/L)	22.00 (17.00,33.00)	18.00 (14.00,27.00)	-7.913	<0.001
UA (umol/L)	303.50 (263.00,355.00)	281.00 (245.00,325.00)	-7.128	<0.001
HbA_1c_ (%)	5.93 ± 0.88	5.75 ± 0.68	4.917	<0.001
FINS (mU/L)	8.05 (5.90,10.70)	6.15 (4.60,8.30)	-11.217	<0.001
FT3 (pmol/L)	4.25 (3.99,4.46)	4.23 (3.99,4.50)	-0.180	0.857
FT4 (pmol/L)	13.04 (12.09,13.93)	13.10 (12.26,14.03)	-1.160	0.246
TSH (mIU/L)	2.04 (1.40,2.86)	2.01 (1.41,2.96)	-0.211	0.833
TPOAb (U/mL)	0.53 (0.27,1.23)	0.47 (0.27,1.14)	-1.168	0.243
TgAb (IU/mL)	1.62 (1.03,10.30)	1.52 (0.96,6.07)	-1.193	0.233

Normal data were represented by mean ± SD, and non-normal data were represented by M *(Q1, Q3).* BMI, body mass index; HOMA-IR, Homeostatic Model Assessment for Insulin Resistance; HC, hip circumference; HR, heart rate; TC, total cholesterol; LDL-C, low-density lipoprotein cholesterol; Scr, serum creatinine; ALT, alanine aminotransferase; AST, aspartate aminotransferase; GGT, gamma-glutamyl transferase; UA, uric acid; HbA1c, glycosylated hemoglobin; FINS, fasting insulin; FT3, free T3; FT4, free T4; TSH, thyroid stimulating hormone; TPOAb, thyroid peroxidase antibody; TgAb, thyroglobulin antibody.

#### 3.1.2 Anthropometric and clinical measurements

The case group exhibited significantly higher values for weight (67.42 ± 9.77 vs 62.39 ± 9.31 kg, *P*<0.001), BMI (25.99 ± 3.36 vs 24.00 ± 3.14 kg/m², *P*<0.001), and HC (100.72 ± 6.47 vs 97.84 ± 6.38 cm, *P*<0.001) compared to controls. (**Table 1**).

#### 3.1.3 Biochemical parameters

The case group demonstrated markedly altered metabolic profiles, including: LDL-C (3.38(2.79,3.96) vs 3.17(2.67,3.71) mmol/L, *P*<0.001), TC (5.54 ± 1.08 vs 5.40 ± 0.97 mmol/L, *P*=0.003).HOMA-IR (2.27 ± 1.19 vs 1.70 ± 0.92, *P*<0.001).GGT (22.00(17.00,33.00) vs 18.00(14.00,27.00) U/L, *P*<0.001), ALT (16.00(13.00,21.00) vs 15.00(11.00,19.00)U/L, *P*<0.001), UA(303.50(263.00,355.00) vs 281.00(245.00,325.00)umol/L, *P*<0.001), HbA1c (5.93 ± 0.88 vs 5.75 ± 0.68%, *P*<0.001), and FINS (8.05(5.90,10.70) vs 6.15(4.60,8.30)mU/L, *P*<0.001). No significant differences were observed in thyroid function tests or other endocrine parameters (*P*>0.05, **Table 1**).

### 3.2 Comparison of lifestyle habits, medical history, and family history between groups

Key lifestyle differences between cases and controls included: Dietary habits: Higher consumption of fresh juice (17.99% vs 13.12%, *P*=0.004),beef and mutton (78.42% vs 74.07%, *P*=0.038), and soda water (20.15% vs 16.32%, *P*=0.049). Physical activity: Lower participation in aerobics (1.20% vs 2.92%, *P*=0.030). Sleep patterns: Shorter average sleep duration (7.55 ± 1.02 vs 7.69 ± 1.17 hours, *P*=0.028). Medical history: Higher prevalence of coronary heart disease (4.86% vs 2.87%, *P*=0.020) and habitual snoring (66.53% vs 54.96%, *P*<0.001). In contrast, no significant intergroup differences emerged in marital status, educational attainment, history of chronic gastroenteritis, smoking, alcohol intake, tea consumption, depression, insomnia, or dietary patterns involving grains, potatoes, pork, poultry, seafood, vegetables, fruits, eggs, milk, soy products, fried items, pickled vegetables, coffee, or animal offal. Measures of physical exertion such as daily vigorous exercise and tai chi, as well as familial predisposition to tumors or diabetes, screen time, and sedentary duration during weekdays, also demonstrated no statistically significant variation (*P* > 0.05) (**Table 2**).

**Table 2:** Comparison of lifestyle habits and different medical histories between groups [n (%)].

Outcome measures	Case group (n=536)	Control group (n=2144)	*χ* ^2^ value	*P value*
Married Status	485 (90.49%)	1949 (90.90%)	0.209	0.648
High School Level or Above	290 (54.10%)	1067 (49.88%)	3.056	0.080
History of Coronary Heart Disease	26 (4.86%)	61 (2.87%)	5.375	0.020
History of Chronic Gastroenteritis	38 (7.12%)	165 (7.75%)	0.246	0.620
Snoring	336 (66.53%)	1102 (54.96%)	22.077	<0.001
Smoking	474 (89.10%)	1915 (89.74%)	0.187	0.665
Alcohol Consumption	388 (72.66%)	1624 (76.21%)	2.908	0.088
Drinking Tea	229 (42.96%)	868 (40.64%)	0.955	0.329
Feeling Depressed	84 (16.00%)	292 (14.04%)	1.306	0.253
Insomnia	138 (26.09%)	579 (27.53%)	0.377	0.539
Grains	533 (99.63%)	1782 (99.50%)	0.145	0.703
Potatoes	510 (95.68%)	2032 (96.17%)	0.262	0.609
Pork	503 (94.55%)	1971 (93.19%)	1.282	0.258
Beef And Mutton	418 (78.42%)	1557 (74.07%)	4.289	0.038
Poultry	407 (76.07%)	1593 (75.75%)	0.025	0.875
Seafood	511 (95.87%)	2017 (95.73%)	0.111	0.739
Vegetables	533 (99.63%)	2100 (99.43%)	0.306	0.580
Fruits	517 (96.82%)	2054 (97.35%)	0.445	0.505
Fresh Juice	95 (17.99%)	433 (13.12%)	8.258	0.004
Eggs	512 (96.06%)	2011 (95.26%)	0.619	0.431
Milk	383 (72.13%)	1543 (73.58%)	0.457	0.499
Soy Products	498 (93.43%)	1982 (93.93%)	0.184	0.668
Fried Food	258 (48.68%)	1018 (48.64%)	0.000	0.987
Soda Water	108 (20.15%)	350 (16.32%)	3.883	0.049
Pickled Vegetables	332 (62.29%)	1280 (60.72%)	0.440	0.507
Coffee	46 (8.66%)	221 (10.56%)	1.666	0.197
Animal Offal	145 (27.31%)	494 (23.57%)	3.216	0.073
Strenuous Exercise	36 (68.70%)	168 (7.95%)	0.682	0.409
Tai Chi	19 (3.54%)	82 (3.82%)	0.079	0.779
Aerobics	6 (1.20%)	59 (2.92%)	4.734	0.030
Family History of Tumor	104 (19.51%)	379 (17.77%)	0.874	0.350
Family History of Diabetes	126 (23.64%)	467 (21.89%)	0.751	0.386
Average Daily Sleep Duration (h)	7.55 ± 1.02	7.69 ± 1.17	-2.198	0.028
Average Daily Television Viewing Time (h)	2.98 ± 1.79	2.92 ± 1.62	0.746	0.456
Time Spent Sitting on Workdays (d)	4.94 ± 0.35	4.90 ± 0.49	1.726	0.085

Dietary habits indicate the consumption of specific foods, while strenuous exercise refers to engagement in intense physical activities within the past seven days. Tai Chi and aerobics assess participation in these exercises over the past 12 months. Snoring reflects whether it occurred during nighttime sleep over the previous year. Depression evaluates depressive feelings within the past two weeks, and insomnia pertains to sleep disturbances during the same period. The time spent sitting on workdays was calculated as the average number of days spent sitting from Monday through Friday.

### 3.3 Multivariate conditional logistic regression analysis of risk factors for MS

Multivariate conditional logistic regression analysis using the forward LR method was employed to investigate risk factors for MS, incorporating variables that demonstrated statistical significance in the univariate analysis. These variables included LDL-C, ALT, GGT, UA, HOMA-IR, HC, BMI, aerobics, consumption of soda water, fresh juice, beef and mutton, average daily sleep duration, history of coronary heart disease, and presence of snoring. Prior to modeling, collinearity diagnostics confirmed the absence of multicollinearity, with VIF values ranging from 1.005 to 1.901, confirming the absence of multicollinearity. The final model identified the following factors remained significantly associated with metabolic syndrome (**Table 3**). Risk factors: LDL-C (OR=1.409, 95%CI 1.245-1.595,*P*<0.001), GGT (OR=1.004, 95%CI 1.001-1.008,*P*=0.017), UA (OR=1.005, 95%CI 1.003-1.007,*P*<0.001), HOMA-IR (OR=1.464, 95%CI 1.313-1.633,*P*<0.001), HC (OR=1.030, 95%CI 1.007-1.053,*P*=0.009), BMI (OR=1.118, 95%CI 1.064-1.174,*P*<0.001), fresh juice consumption (OR=1.846, 95%CI 1.315-2.592,*P*<0.001), and beef and mutton intake (OR=1.282, 95%CI 1.007-1.632,*P*=0.044).Protective factor: Longer sleep duration (OR=0.844, 95%CI 0.761-0.936,*P*=0.001). Among them, LDL-C showed the strongest positive association (41% increased odds per unit), fresh juice consumption conferred the highest modifiable risk (85% increased odds), sleep duration emerged as the most robust protective factor (16% risk reduction per hour).

**Table 3:** Multivariate conditional logistic regression analysis.

Variables	β value	SE value	Wald*χ* ^2^ value	*P* value	OR value	95%CI
LDL-C	0.343	0.063	29.525	<0.001	1.409	1.245-1.595
ALT	-0.003	0.006	0.324	0.569	0.997	0.986-1.008
GGT	0.004	0.002	5.695	0.017	1.004	1.001-1.008
UA	0.005	0.001	26.661	<0.001	1.005	1.003-1.007
HOMA-IR	0.381	0.056	46.899	<0.001	1.464	1.313-1.633
HC	0.029	0.011	6.747	0.009	1.030	1.007-1.053
BMI	0.112	0.025	19.891	<0.001	1.118	1.064-1.174
Aerobics	-0.620	0.475	1.703	0.192	0.538	0.212-1.365
Soda Water	-0.073	0.155	0.224	0.636	0.929	0.686-1.259
Fresh Juice	0.613	0.173	12.541	<0.001	1.846	1.315-2.592
Beef and Mutton	0.248	0.123	4.057	0.044	1.282	1.007-1.632
Snoring	-0.072	0.048	2.295	0.130	0.930	0.847-1.021
Average Daily Sleep Duration	-0.170	0.053	10.316	0.001	0.844	0.761-0.936
History of Coronary Heart Disease	0.476	0.320	2.208	0.137	1.609	0.859-3.015

Aerobic exercise indicates engagement in aerobics within the past 12 months. Dietary habits denote the frequency of specific food consumption. Snoring refers to the occurrence of snoring during nighttime sleep over the past 12 months.

## 4 Discussion

The primary endpoint in this study was MS. Statistically significant variables identified through univariate analysis—including LDL-C, ALT, GGT, UA, HOMA-IR, HC, BMI, engagement in aerobics, soda and fresh juice intake, consumption of beef and mutton, average daily sleep duration, history of coronary heart disease, and snoring—were entered into a multivariate conditional logistic regression model using the forward LR method. The analysis revealed that longer average sleep duration as a protective factor against MS risk. Conversely, elevated levels of LDL-C, GGT, UA, HOMA-IR, HC, and BMI, along with consumption of fresh juice and red meat (beef and mutton), were significantly associated with increased MS risk. Among the modifiable behavioral variables, average sleep duration demonstrated an inverse association with MS, whereas fresh juice and red meat(beef and mutton) consumption exhibited positive associations.

### 4.1 Sleep duration

Notably, our cohort exhibited shorter average sleep durations (7.55h in cases vs. 7.69h in controls), reflecting Dalian’s fast-paced urban lifestyle. Current investigations into the association between sleep duration and MS yield inconsistent outcomes. One meta-analysis identified a U-shaped relationship, indicating increased MS risk at both extremes of sleep duration (15). In contrast, data from the China Health and Retirement Longitudinal Study revealed that sleep exceeding 8 h/d was linked to a 53% reduction in MS incidence compared to the 7–8 h/d reference group (16). A more recent meta-analysis including 11 studies with 343,669 participants found a higher MS prevalence among individuals reporting normal sleep duration than among those with either short or extended sleep durations. Regionally, North America exhibited the highest MS prevalence among both short and long sleepers, whereas in Asia, the highest rates were noted among those with typical sleep durations (17). No analogous research has been conducted in Dalian. Findings from the current analysis suggest that average daily sleep duration may exert a protective effect against MS. Potential mechanisms underlying this association include the synthesis and release of melatonin, which primarily occur at night and are inhibited by daytime light exposure. Melatonin exerts lipid-lowering, anti-inflammatory, and antioxidant effects, while also regulating blood pressure (18). Research (19) has identified significant differences in nocturnal melatonin secretion between individuals with and without MS, with disruptions in circadian melatonin rhythms associated with MS onset. Additionally, MS patients exhibit heightened sympathetic nervous system activity (20). Reduced sleep duration, combined with elevated sympathetic drive, contributes to the development of hypertension (21). Sympathetic activation stimulates lipolysis through adipose tissue innervation, increasing circulating free fatty acids, which in turn diminishes insulin sensitivity and impair glucose tolerance (22, 23). Further evidence (24) also indicates that sleep deprivation influences hormones governing appetite and eating behavior, promoting increased food intake and subsequent weight gain, thereby predisposing to overweight and obesity. In parallel, reduced sleep duration has been shown to upregulate proinflammatory mediators (25, 26), which promote insulin resistance in both adipose and peripheral tissues (27), further increasing susceptibility to MS. Collectively, these mechanisms collectively explain our observed association between average daily sleep duration and MS. Future studies should assess sleep quality and napping habits, as Dalian residents rarely nap because of its short lunch breaks, potentially compounding sleep-related metabolic risks. Given the protective role of sleep duration, community-based initiatives could raise awareness about the importance of adequate sleep and provide practical tips for improving sleep duration, such as reducing screen time before bed, creating sleep-conducive environments, increasing the lunch break time.

### 4.2 Fresh juice consumption

In the questionnaire of this research, the definition of fresh juice is “juice extracted from fresh fruits”, without any additional additives or processing procedures. Current evidence regarding the metabolic impact of fresh juice consumption remains inconsistent. Our finding that fresh juice intake increases MS risk contrasts with a cohort study reporting protective effects of pure fruit juice (28). This discrepancy may arise from differences in juice composition and consumption patterns. Conversely, other studies (29) align with the present findings, indicating a positive correlation between fresh juice consumption and MS development. In our study, “fresh juice” likely contains high in fructose but low in fiber. Unlike whole fruits, juicing removes dietary fiber, accelerating fructose absorption (30). Fructose undergoes hepatic metabolism distinct from that of glucose. In the absence of a rate-limiting enzyme and feedback inhibition, fructose catabolism yields high levels of uric acid, diglycerides, lactic acid, and other intermediates, which may trigger endoplasmic reticulum stress and inflammatory responses. These byproducts interfere with key metabolic pathways, promoting insulin resistance, lipogenesis, vascular endothelial impairment, central adiposity, elevated triglyceride concentrations, decreased HDL-C, hypertension, and impaired glucose tolerance—core features of MS. Furthermore, fructose modulates gut microbiota composition and activity (31), and the gut microbiota and metabolites have been proven to increase the risk of diabetes, metabolism-related fatty liver disease, carotid atherosclerotic plaque and MS (32, 33). Notably, Dalian’s warm climate and abundant fruit markets may encourage frequent juice consumption, exacerbating these effects. Thus, public health campaigns in Dalian should emphasize whole fruit consumption over juicing, particularly among high-risk groups.

### 4.3 Red meat (beef and mutton) consumption

The results of this study align with previous research (34), indicating that the consumption of beef and mutton (red meat) may heighten the risk of MS. Although red meat essential nutrients such as amino acids, vitamins, and minerals (e.g., iron and zinc), growing evidence links its intake to an increased risk of various chronic diseases. Several biological pathways may account for the observed relationship between red meat consumption and MS development. One proposed mechanism involves the high heme iron content in beef and mutton, which functions as a potent pro-oxidant. Excessive intake of heme iron promotes oxidative stress, thereby triggering cellular damage and chronic systemic inflammation (35). Moreover, the processing and cooking techniques commonly applied to red meat appear to enhance its harmful metabolic effects (36). In Dalian, longstanding dietary practices such as hot pot and street barbecue are culturally ingrained, with beef and mutton as central ingredients. During these high-temperature cooking processes, significant levels of nitrates and nitrites are generated, which have been implicated in the induction of insulin resistance (37), potentially increasing susceptibility to MS. Additionally, the elevated content of total fat and saturated fatty acids in beef and mutton contributes to obesity, hyperinsulinemia, and hyperglycemia, exacerbate insulin resistance and further contributing to the onset of MS (38). Studies have also shown elevated levels of inflammatory mediators in individuals who regularly consume beef and mutton, and processed meats, potentially explaining the heightened risk of MS in this population (39). A longstanding belief in Dalian attributes tonic and restorative properties to the consumption of beef and mutton, and their broths, particularly mutton soup, which remains popular among locals. Although beef and mutton consumption is deeply embedded in Dalian’s culinary culture, its association with MS calls for strategies to mitigate metabolic harm. For example, co-administration of compounds like Xiasangju, a traditional Chinese herbal formula, may attenuate red meat-induced oxidative stress and inflammation. Studies suggest that Xiasangju’s noradrenaline-enhancing properties can activate brown adipose tissue, thereby increasing energy dissipation and improving lipid profiles (40). This synergistic approach that combines dietary factors that promote the occurrence of MS with those protect it could be explored in future public health campaigns.

### 4.4 Biomarkers: LDL-C, GGT, UA, HOMA-IR, HC, and BMI

Consistent with most previous studies, elevated LDL-C, GGT, UA, HOMA-IR, HC, and BMI are identified as significant indicators for increased risk of MS (41–43). LDL-C contributes to atherosclerosis by depositing oxidized lipids in arterial walls, while GGT, a marker of hepatic steatosis, reflects systemic oxidative stress (44). A recent study highlighted the differences in the effects of lipids and lipoproteins on BP and pulse pressure. For pulse pressure, the dangerous effect of LDL-C bears the brunt among the major lipids (45). UA in both crystalline and soluble forms, plays a key role in the induction of inflammatory cascade and development of atherosclerotic diseases (46). HOMA-IR and HC underscore the centrality of insulin resistance and central obesity in MS pathogenesis. The increase of BMI drives higher ratio of 12,13-Epoxyoctadecenoic acid: Dihydroxyoctadecenoic acid in white adipose tissue and liver, which indicates the deterioration of the MS (47). Notably, Dalian’s rapid urbanization has likely amplified sedentary behaviors and energy-dense diets, exacerbating these biomarkers. Clinicians should prioritize these metrics in routine screenings to enable early MS detection.

This study’s nested case-control design enhances efficiency and reduces recall bias compared to traditional case-control studies. However, several methodological limitations warrant careful consideration regarding their potential impact on the results. First, possibility of residual confounding or the influence of unmeasured variables (e.g., sample contamination, diet before blood collection, impact of a woman’s menstrual period, socioeconomic status, dietary additives, or environmental pollutants, etc.) cannot be ruled out. Second, the reliance on self-reported dietary data may introduce recall bias, particularly given the 3-year interval between baseline and follow-up. Third, due to participants’ limited recall accuracy and over 50% missing data for portion size, analysis involving frequency and quantity is excluded. A binary variable (yes/no) is adopted for statistical modeling, potentially masking thresholds at which fresh juice or red meat intake becomes clinically significant. Moreover, the questionnaire does not differentiate cooking methods for beef and mutton or specify the types and preparation techniques of fresh juice. Finally, while the study adjusted for key confounders (e.g., age, sex), the absence of longitudinal assessments limits causal inference. For example, the association between short sleep duration and MS might be bidirectional, as MS-related metabolic disturbances could also disrupt sleep. Despite these limitations, the consistency of our findings with prior mechanistic research supports their biological plausibility.

The present study reveals a significant correlation between the occurrence of MS in adult residents of Dalian and several factors, including elevated levels of LDL-C, GGT, UA, HOMA-IR, HC, and BMI, as well as reduced daily sleep duration, consumption of beef and mutton, and intake of fresh juice. These results align with some existing literature but also underscore the need for targeted interventions and further research to address these factors in the Dalian population. Future research should employ longitudinal designs to establish causal relationships between identified risk factors and MS. For example, tracking changes in dietary habits, sleep patterns, and biomarker levels over time could elucidate their long-term impact on MS development, providing stronger evidence for causality and inform public health strategies. In addition, targeted public health campaigns should be carried out, such as providing targeted dietary advice, strengthening publicity on the importance of sleep, and launching projects for regular monitoring of relevant biological indicators in community hospitals. By addressing dietary habits, sleep duration, and biomarker monitoring, Dalian might reduce the burden of MS and improve overall metabolic health.

## Data Availability

The data analyzed in this study is subject to the following licenses/restrictions: The raw data from REACTION’s study has not yet been released. Requests to access these datasets should be directed to 18840859380@163.com.
